# Combination of C-Reactive Protein and Neutrophil-to-Lymphocyte Ratio as a Novel Prognostic Index in Patients With Bladder Cancer After Radical Cystectomy

**DOI:** 10.3389/fonc.2021.762470

**Published:** 2021-12-02

**Authors:** Yidi Wang, Keyi Wang, Jinliang Ni, Houliang Zhang, Lei Yin, Yifan Zhang, Huajuan Shi, Tao Zhang, Naichun Zhou, Weipu Mao, Bo Peng

**Affiliations:** ^1^ Department of Urology, Shanghai Putuo District People’s Hospital, Tongji University, Shanghai, China; ^2^ Department of Urology, Shanghai Tenth People’s Hospital, School of Medicine, Tongji University, Shanghai, China; ^3^ Shanghai Clinical College, Anhui Medical University, Hefei, China; ^4^ Department of Urology, Xinyang Central Hospital, Xinyang, China; ^5^ Department of Urology, Affiliated Zhongda Hospital of Southeast University, Nanjing, China

**Keywords:** bladder cancer, radical cystectomy, C-reactive protein, neutrophil-to-lymphocyte ratio, prognosis, nomogram

## Abstract

**Background:**

Inflammation is widely considered an important hallmark of cancer and associated with poor postoperative survival. The objective of this study is to assess the significance of preoperative C-NLR, a new inflammation-based index that includes preoperative C-reactive protein (CRP) and neutrophil-to-lymphocyte ratio (NLR), on therapeutic outcomes for bladder cancer (BC) patients after radical cystectomy (RC).

**Materials and Methods:**

BC patients who underwent RC between 2010 and 2019 were retrospectively analyzed from our medical center. The predictive effect of CRP, NLR, and C-NLR on the survival of BC patients were analyzed by the receiver operating characteristic (ROC) curves. The relationship between C-NLR and postoperative survival was investigated by Cox regression. The corresponding nomograms were built based on the Cox regression results of overall survival (OS) and disease-free survival (DFS), which were further validated by ROC curves, decision curve analysis (DCA) curves, and calibration curves.

**Results:**

Of the 199 eligible patients, 83 (41.70%) were classified as high C-NLR group and the remaining 116 (58.30%) were classified as low C-NLR group. ROC analysis showed that C-NLR had the largest area under curve (AUC) compared to CRP and NLR. Multivariate analysis revealed that T-stage and C-NLR [high C-NLR vs. low C-NLR, hazard ratio (HR) = 2.478, 95% confidence interval (CI), 1.538–3.993, *p* < 0.001] were independent predictors of OS, whereas T-stage, M-stage, and C-NLR (high C-NLR vs. low C-NLR, HR = 2.817, 95% CI, 1.667–4.762, *p* < 0.001) were independent predictors of DFS. ROC and DCA analysis demonstrated better accuracy and discrimination of 3- and 5-year OS and DFS with C-NLR-based nomogram compared to TNM stage. The calibration curve reconfirmed the accurate predicting performance of nomograms.

**Conclusion:**

C-NLR is a reliable predictor of long-term prognosis of BC patients after RC and will contribute to the optimization of individual therapy for BC patients.

## Introduction

Bladder cancer (BC) is one of the most common malignant tumors worldwide, with 9th morbidity and 13th mortality rate among malignant tumors, respectively (1). According to the TNM stage, BC can be categorized as non-muscular invasive bladder cancer (NMIBC) and muscular invasive bladder cancer (MIBC). Currently, radical cystectomy (RC) with regional pelvic lymphadenectomy (PLND) is the established standard therapy for MIBC and high-risk NMIBC (2, 3). However, the survival rate of patients after RC is not satisfactory, with 5-year and 10-year overall survival (OS) rates of 66% and 43%, respectively (4). In addition to the fact that RC is a high-risk procedure, BC is a heterogeneous group of tumors with at least 40 histological subgroups, which greatly increases the complexity of its management and prognosis (5). Furthermore, the lack of reliable prognostic indicators after RC hinders individual therapy and somehow exposes patients to overtreatment or undertreatment. Therefore, it is essential to search robust prognostic biomarkers for patients who underwent RC to improve the OS of BC patients.

To date, numerous reports have confirmed that inflammation as a hallmark of cancer substantially contributes to the development and progression of malignancies (6, 7). Based on this knowledge, a number of studies have revealed the validity of systemic inflammatory markers such as albumin, neutrophils, lymphocytes, platelets, C-reactive protein (CRP), and also biomarker combination ratios [e.g., neutrophil–lymphocyte ratio (NLR), platelet–lymphocyte ratio (PLR), and CRP–albumin ratio (CAR)] as predictive factors in different cancers (8, 9). These markers are easy to obtain compared to invasive tests and have shown some value in individualized clinical treatment. Alternatively, it still lacks recognized indicators with significant prognostic value in patients with BC who underwent RC. Recently, a new index, C-NLR, which consists of both CRP and NLR, has been proposed, and its prognostic significance was validated in patients with pancreatic cancer after pancreatic resection (10). In view of the above reasons, we initially evaluated the prognostic value of C-NLR in BC patients undergoing RC in this study.

## Patients and Methods

### Patients

A total of 199 retrospective medical records of BC patients who underwent RC at the Department of Urology, Shanghai Tenth People’s Hospital from February 2010 to January 2019 were obtained. The inclusion criteria for patients were as follows: (1) patients who underwent RC; and (2) postoperative pathological diagnosis clearly confirmed BC. Exclusion criteria were as follows: (1) patients with a combined history of other malignancies; (2) patients with hematologic and autoimmune diseases; (3) patients who received radiotherapy or chemotherapy within 1 month preoperatively; (4) incomplete clinical or laboratory data; and (5) incomplete follow-up data. This study was approved by the Ethics Committee of the First Affiliated Hospital of Zhengzhou University before the start of the study.

### Clinical Variables

The following patient characteristics were obtained for analysis: age at surgery, gender, body mass index (BMI), adjuvant chemotherapy or not, baseline TNM classification (according to the 8th edition of the TNM stage), and tumor grade (tumor grading and staging were based on clinicopathological reports). Preoperative laboratory test results within 3 days such as neutrophils, lymphocytes, and CRP were collected and used to calculate NLR and C-NLR. NLR was calculated as neutrophil count divided by lymphocyte count, and C-NLR was calculated as serum CRP level (mg/L) × NLR.

### Patient Follow-Up

Each patient included in this study was followed up regularly after surgery. Follow-up was performed every 3 months for the first 2 years after RC, every 6 months in the third year, and annually thereafter. The last survival follow-up date was January 31, 2020. The endpoint of follow-up for each patient was the final follow-up date or the patient’s death, and if death occurred, the time and cause of death were recorded in detail. OS was calculated from receipt of RC to death or to the date of the last follow-up. Disease-free survival (DFS) was calculated from receipt of RC to disease recurrence or to death due to BC disease progression or to the date of the last follow-up.

### Statistical Analysis

In this study, all statistical analyses were performed using SPSS v24.0 (SPSS Inc, Chicago, IL, USA), and all continuous variables were expressed as interquartile range (IQR). Based on professional knowledge, the results collected from the disease and reference groups were analyzed to determine the upper and lower limits of measurement values, group spacing and cutoff points, and the cumulative frequency distribution tables were listed according to the selected group spacing intervals, and the sensitivity, specificity, and false-positive rates (1 − specificity) were calculated for all cutoff points, respectively. The sensitivity was used as the vertical coordinate to represent the true-positive rate, and (1 − specificity) was used as the horizontal coordinate to represent the false-positive rate, which was plotted as receiver operating characteristic (ROC) curve by Medcalc (version 15.2.2.0, Ostend, Belgien) (11). CRP, NLR, and C-NLR optimal cutoff values were determined by Medcalc based on ROC curves. In this study, true positive was defined as a post-RC BC patient who was predicted to die and did die in the actual situation. False positive was defined as a post-RC BC patient who was predicted to die but did not die in the actual situation. OS and DFS were compared by Kaplan–Meier survival analysis in accordance with each cutoff value, and log-rank tests were applied to test the differences. ROC curves for CRP, NLR, and C-NLR were conducted and the area under the curves (AUC) were compared. Univariate and multivariate Cox regression models were employed to analyze potential associated risk factors, with *p*-values <0.05 in the univariate analysis included in the multivariate analysis, and backward stepwise selection was used to identify independent factors associated with OS and DFS. Based on the multivariate Cox analysis results, nomograms were generated in R 3.2.1 (Institute for Statistics and Mathematics, Vienna, Austria) software. Decision curve analysis (DCA) and ROC curves were applied to evaluate and compare the predictive performance of nomograms and TNM stage. Calibration curves were used to assess the accuracy of the nomograms, and the results indicate perfect prediction accuracy when calibration curves were consistent with the 45° line. *p*-values < 0.05 were considered statistically significant.

## Results

### Patient Characteristics

Total 199 BC patients who met the inclusion criteria were enrolled in this study. The median age was 66.00 years with an IQR 60.00–73.00 years, and 174 of them were males. Using the eighth edition TNM stage, 79 patients (39.4%) were classified as T1, 42 (21.2%) as T2, 40 (20.2%) asT3, and 38 (19.2%) as T4. Low-grade tumors were present in 10 patients (5.9%) and 189 (94.1%) had high-grade tumors. The medians of CRP, neutrophil, and lymphocyte were 4.70 g/L, 4.95 × 10^9^/L, and 1.60 × 10^9^/L respectively, and their respective IQRs were 3.27–25 × 10 g/L, 3.38–7.24 × 10^9^/L, and 1.20–2.12 × 10^9^/L. The medians of NLR and C-NLR were calculated as 3.12 and 15.94 with IQRs of 2.02–5.91 and 6.62–115.25. As for the number of lymph nodes retrieved and number of positive lymph nodes retrieved, the medians were both 2.00 and the IQRs were 1.00–5.00 and 1.00-4.00, respectively. The number of patients with positive lymph nodes retrieved was 31. No patients have positive soft tissue surgical margins. The median follow-up was 20.00 months with an IQR of 10.00–49.00 months. There were 125 patients who survived at the end point of follow-up, with an OS rate of 62.8% and a DFS rate of 68.3%. Additional clinicopathological characteristics of the patients are summarized in **Table 1**.

**Table 1:** Baseline characteristics of included BC patients.

Variables	*n* (%)
Total patients	199 (100.0)
Age, [years, median (IQR)]	66.00 (60.00, 73.00)
Age categorized	
≤67	113 (56.6)
>67	86 (43.4)
Gender	
Male	174 (86.7)
Female	25 (13.3)
BMI, [kg/m^2^, median (IQR)]	23.05 (21.26, 25.24)
BMI categorized, (kg/m^2^)	
<18.5	10 (4.9)
18.5–23.9	111 (54.1)
24.0–26.9	53 (26.6)
≥27.0	25 (12.8)
Adjuvant chemotherapy	
No	174 (86.6)
Yes	25 (13.4)
pT-stage	
T1	79 (39.4)
T2	42 (21.2)
T3	40 (20.2)
T4	38 (19.2)
pN-stage	
N0	166 (82.7)
N1	33 (17.3)
pM-stage	
M0	192 (95.5)
M1	7 (4.5)
Grade	
Low grade	10 (5.9)
High grade	189 (94.1)
CRP, [g/L, median (IQR)]	4.70 (3.27, 25.10)
Neutrophil, [10^9^, median (IQR)]	4.95 (3.38, 7.24)
Lymphocyte, [10^9^, median (IQR)]	1.60 (1.20, 2.12)
NLR, median (IQR)	3.12 (2.02, 5.91)
C-NLR, median (IQR)	15.94 (6.62, 115.25)
Number of lymph nodes retrieved [*n*, median (IQR)]	2.00 (1.00, 5.00)
Number of patients with positive lymph nodes retrieved, *n* (%)	31 (15.6%)
Number of positive lymph nodes retrieved [*n*, median (IQR)]	2.00 (1.00, 4.00)
Positive soft tissue surgical margins (*n*)	0
Follow-up [months, median (IQR)]	20.00 (10.00, 49.00)
Overall survival, *n* (%)	125 (62.8%)
Disease-free survival, *n* (%)	136 (68.3%)

IQR, Interquartile range; BC, Bladder cancer; BMI, Body mass index; AJCC, American Joint Committee on Cancer; CRP, C-reactive protein; NLR, neutrophil-to-lymphocyte ratio.

**Figure 1:**
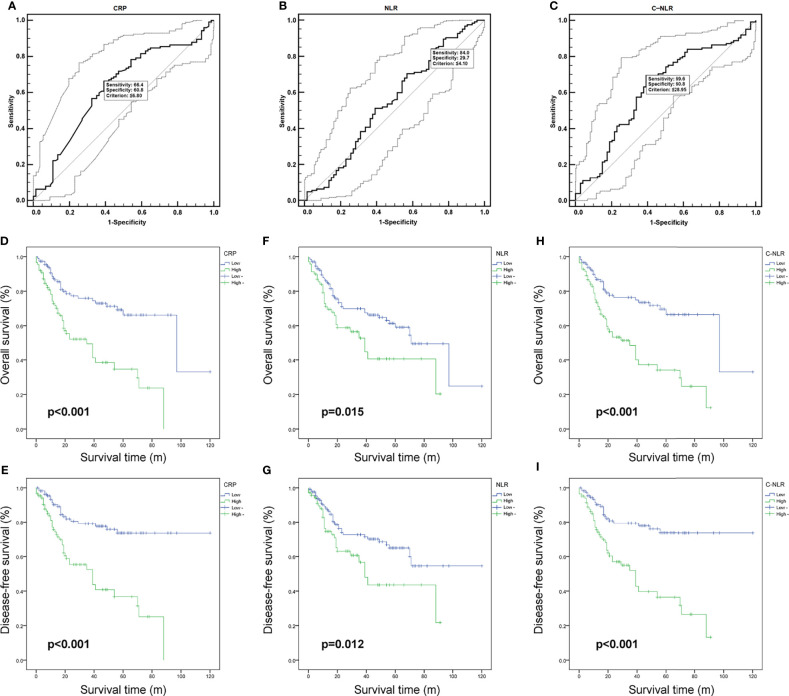
The receiver operating characteristic (ROC) curves of C-reactive protein (CRP) **(A)**, neutrophil-to-lymphocyte ratio (NLR) **(B)**, and C-NLR **(C)**. Kaplan–Meier curves for overall survival (OS) and disease-free survival (DFS) after radical cystectomy stratified by CRP **(D, E)**, NLR **(F, G)**, and C-NLR **(H, I)**.

The optimal cutoff values for CRP, NLR, and C-NLR determined by ROC analyses were as follows: 6.80, 4.10, and 28.95 (**Figures 1A–C**). Patients were divided into high and low groups for further analysis based on the optimal cutoff values. **Table 2** summarized the clinicopathological characteristics of the patients based on CRP and NLR. After CRP-based stratification, 112 (59.82%) patients were classified in the CRP-low group, which had a younger age than the CRP-high group (64.45 ± 9.69 vs. 68.10 ± 10.29; *p* = 0.016). Moreover, the proportion of patients with stage N1 was lower in the CRP-low group (10.7%) than in the CRP-high group (24.1%). As for NLR-based stratification, there are 130 (65.3%) patients in the low group and 69 (34.7%) patients in the high group.

**Table 2:** Baseline characteristics according to the subgroups classified by CRP and NLR.

Characteristic	CRP		*p-*value	NLR		*p-*value
	Low	High		Low	High	
	*n* (%)	*n* (%)		*n* (%)	*n* (%)	
Total patients	112 (40.17)	87 (59.82)		130 (65.30)	69 (34.70)	
Age, [years, median (IQR)]	64.00 (59.25, 70.00)	69.00 (60.00, 75.00)	0.016	65.00 (59.00, 72.00)	70.00 (63.00, 79.00)	0.082
Age categorized			0.001			0.031
≤67	75 (67.0)	38 (43.7)		81 (62.3)	32 (46.4)	
>67	37 (33.0)	49 (56.3)		49 37.7)	37 (53.6)	
Gender			0.737			0.144
Male	96 (85.7)	76 (87.4)		109 (83.8)	63 (91.3)	
Female	16 (14.3)	11 (12.6)		21 (16.2)	6 (8.7)	
BMI, [kg/m^2^, median (IQR)]	23.77 (21.80, 25.60)	22.51 (20.90, 24.77)	0.128	23.39 (21.74, 25.71)	22.31 (20.72, 23.83)	0.388
BMI categorized, (kg/m^2^)			0.001			0.006
<18.5	4 (3.6)	7 (8.0)		6 (4.6)	5 (7.2)	
18.5–23.9	54 (48.2)	55 (63.2)		61 (46.9)	48 (69.6)	
24.0–26.9	41 (36.6)	11 (12.6)		41 (31.5)	11 (15.9)	
≥27.0	13 (11.6)	14 (13.6)		22 (16.9)	5 (7.2)	
Adjuvant chemotherapy			0.182			0.781
No	100 (89.3)	72 (82.8)		113 (86.9)	59 (85.5)	
Yes	12 (10.7)	15 (17.2)		17 (13.1)	10 (14.5)	
pT-stage			0.184			0.638
T1	48 (42.9)	30 (34.5)		49 (37.7)	29 (42.0)	
T2	27 (24.1)	15 (17.2)		31 (23.8)	11 (15.9)	
T3	19 (17.0)	22 (25.3)		26 (20.0)	15 (21.7)	
T4	18 (16.1)	20 (23.0)		24 (18.5)	14 (20.3)	
pN-stage			0.012			0.154
N0	100 (89.3)	66 (75.9)		112 (86.2)	54 (78.3)	
N1	12 (10.7)	21 (24.1)		18 (13.8)	15 (21.7)	
pM-stage			0.715			0.452
M0	108 (96.4)	83 (95.4)		126 (96.9)	65 (94.2)	
M1	4 (3.6)	4 (4.6)		4 (3.1)	4 (5.8)	
Grade			0.882			1.000
Low grade	7 (6.2)	5 (5.7)		8 (6.2)	4 (5.8)	
High grade	105 (93.8)	82 (94.3)		122 (93.8)	65 (94.2)	

CRP, C-reactive protein; NLR, neutrophil-to-lymphocyte ratio; SD, standard deviation; BMI, Body mass index; AJCC, American Joint Committee on Cancer.

Furthermore, we compared patients in the low and high groups to investigate the clinical characterization based on C-NLR in **Table 3**. There are 116 (58.30%) and 83 (41.70%) patients in the C-NLR-low group and C-NLR-high group, respectively. The results revealed that patients with lower C-NLR levels were further likely to have a higher BMI level (24.03 ± 2.83 vs. 22.51 ± 3.42; *p* = 0.001) and to be younger (64.59 ± 9.38 vs. 68.33 ± 10.83; *p* = 0.010) compared with those with higher C-NLR levels.

**Table 3:** Baseline characteristics according to the subgroups classified by C-NLR.

Characteristic	C-NLR		*p-*value
	Low	High	
	*n* (%)	*n* (%)	
Total patients	116 (58.30)	83 (41.70)	
Age, [years, median (IQR)]	63.50 (58.25, 70.00)	69.00 (64.00, 76.00)	0.010
Age categorized			<0.001
≤67	79 (68.1)	34 (41.0)	
>67	37 (31.9)	49 (59.0)	
Gender			0.678
Male	99 (85.3)	73 (88.0)	
Female	17 (14.7)	10 (12.0)	
BMI, [kg/m^2^, median (IQR)]	23.93 (22.21, 25.85)	22.40 (20.41, 24.46)	0.001
BMI categorized, (kg/m^2^)			<0.001
<18.5	3 (2.6)	8 (9.6)	
18.5–23.9	54 (46.6)	55 (66.3)	
24.0–26.9	41 (35.3)	11 (13.3)	
≥27.0	18 (15.5)	9 (10.8)	
Adjuvant chemotherapy			0.117
No	104 (89.7)	68 (81.9)	
Yes	12 (10.3)	15 (18.1)	
pT-stage			0.554
T1	48 (41.4)	30 (36.1)	
T2	27 (22.4)	16 (19.3)	
T3	20 (17.2)	21 (25.3)	
T4	22 (19.0)	16 (19.3)	
pN-stage			0.102
N0	101 (87.1)	65 (78.3)	
N1	15 (12.9)	18 (21.7)	
pM-stage			0.721
M0	112 (96.6)	79 (95.2)	
M1	4 (3.4)	4 (4.8)	
Grade			0.998
Low grade	7 (6.0)	5 (6.0)	
High grade	109 (94.0)	78 (94.0)	

SD, standard deviation; BMI, Body mass index; AJCC, American Joint Committee on Cancer.

### Survival Analyses Based on CRP, NLR, and C-NLR

**Figure 2:**
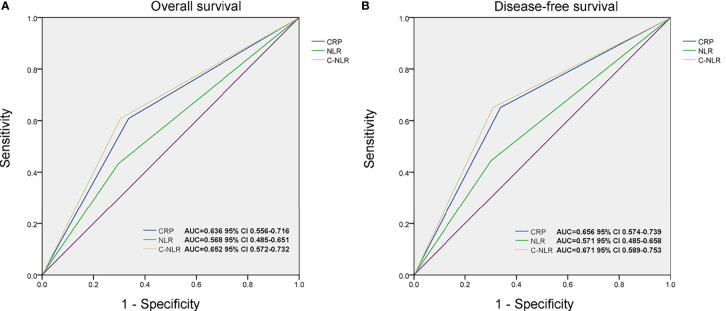
The area under the curves (AUCs) of C-reactive protein (CRP), neutrophil-to-lymphocyte ratio (NLR), and C-NLR in overall survival (OS) **(A)** and disease-free survival (DFS) **(B)** prediction after radical cystectomy were compared based on the results of receiver operating characteristic (ROC) analyses.

According to CRP, NLR, and C-NLR, the Kaplan–Meier survival curves were conducted for OS and DFS in **Figure 1**. The Kaplan–Meier survival analysis demonstrated that low levels of CRP (≤6.8 mg/L), NLR (≤4.10), and C-NLR (≤28.95) were all significantly associated with better OS (*p* < 0.001, *p* = 0.015, and *p* < 0.001; **Figures 1D, F, H**) and DFS (*p* < 0.001, *p* = 0.012, and *p* < 0.001; **Figures 1E, G, I**). ROC analysis provided further insight into the prognostic value of these three factors in **Figure 2**. As shown in the ROC analysis, the AUCs of C-NLR were 0.652 and 0.671 for OS and DFS, which were distinctly larger than CRP and NLR (**Table 4**). This suggested that the prognostic value of C-NLR is the best of these three.

**Table 4:** The AUCs of CRP, NLR, and C-NLR for OS and DFS prediction based on the ROC results.

Characteristics	OS			DFS		
	AUC	95% CI	p-value	AUC	95% CI	p-value
CRP	0.636	0.556–0.716	0.001	0.656	0.574–0.739	<0.001
NLR	0.568	0.485–0.651	0.108	0.571	0.485–0.658	0.105
C-NLR	0.652	0.572–0.732	<0.001	0.671	0.589–0.753	<0.001

AUC, area under curve; CRP, C-reactive protein; NLR, neutrophil-to-lymphocyte ratio; OS, overall survival; DFS, disease-free survival; ROC, receiver operator characteristic; CI, confidence interval.

### Factors Associated With OS and DFS

Univariate analysis and multivariate analyses were performed to identify parameters associated with OS and DFS. On univariate analysis, age, T-stage, N-stage, M-stage, CRP, NLR, and C-NLR were significantly associated with OS after RC (**Table 5**). On multivariate analysis, only T stage (*p* < 0.001) and C-NLR (*p* < 0.001) were independent predictors of OS after RC (**Table 5**). For DFS, univariate analyses demonstrated that age, T-stage, N-stage, M-stage, CRP, NLR, and C-NLR were associated with the DFS of BC patients who underwent RC (**Table 6**). Multivariate analyses of each factor with *p* < 0.05 in univariate analyses revealed that T-stage, M-stage, and C-NLR were independent predictors of OS (**Table 6**).

**Table 5:** Univariate and multivariate analyses of factors associated with overall survival (OS).

Characteristics	Univariate analysis		Multivariate analysis^a^	
	HR (95% CI)	*p*-value	HR (95% CI)	*p*-value
Age categorized, years				
≤67	Reference		Reference	
>67	1.873 (1.182–2.967)	0.008	–	0.187
Gender				
Male	Reference			
Female	1.013 (0.519–1.977)	0.971		
Chemotherapy				
No	Reference			
Yes	1.512 (0.856–2.669)	0.154		
T-stage				
T1	Reference		Reference	
T2	2.127 (1.021–4.432)	0.044	2.076 (0.997–4.320)	0.051
T3	3.806 (1.963–7.381)	<0.001	3.519 (1.812–6.834)	<0.001
T4	5.577 (2.867–10.848)	<0.001	5.139 (2.646–9.982)	<0.001
N-stage				
N0	Reference		Reference	
N1	3.331 (2.002–5.544)	<0.001	–	0.129
M-stage				
M0	Reference		Reference	
M1	3.032 (1.217–7.551)	0.017	–	0.084
Grade				
Low grade	Reference			
High grade	1.885 (0.677–5.250)	0.225		
CRP				
Low	Reference		Reference	
High	2.814 (1.746–4.534)	<0.001	–	0.392
NLR				
Low	Reference		Reference	
High	1.770 (1.112–2.819)	0.016	–	0.488
C-NLR				
Low	Reference		Reference	
High	2.723 (1.691–4.386)	<0.001	2.478 (1.538–3.993)	<0.001

HR, Hazard ratio; CI, Confidence interval; AJCC, American Joint Committee on Cancer; CRP, C-reactive protein; NLR, neutrophil-to-lymphocyte ratio.

a Adjusted to age, T-stage, N-stage, M-stage, CRP, NLR, and C-NLR.

**Table 6:** Univariate and multivariate analyses of factors associated with disease-free survival (DFS).

Characteristics	Univariate analysis		Multivariate analysis^a^	
	HR (95% CI)	*p*-value	HR (95% CI)	*p*-value
Age categorized, years				
≤67	Reference		Reference	
>67	1.795 (1.092–2.951)	0.021	–	0.580
Gender				
Male	Reference			
Female	0.928 (0.441–1.951)	0.843		
Chemotherapy				
No	Reference			
Yes	1.670 (0.921–3.027)	0.091		
T-stage				
T1	Reference		Reference	
T2	3.836 (1.584–9.288)	0.003	3.779 (1.561–9.150)	0.003
T3	6.704 (2.958–15.192)	<0.001	6.086 (2.680–13.821)	<0.001
T4	9.630 (4.211–22.020)	<0.001	8.074 (3.497–18.639)	<0.001
N-stage				
N0	Reference		Reference	
N1	3.664 (2.133–6.295)	<0.001	–	0.116
M-stage				
M0	Reference		Reference	
M1	3.696 (1.475–9.260)	0.005	2.668 (1.031–6.903)	0. 043
Grade				
Low grade	Reference			
High grade	2.006 (0.626–6.432)	0.242		
CRP				
Low	Reference		Reference	
High	3.297 (1.952–5.567)	<0.001	–	0.282
NLR				
Low	Reference		Reference	
High	1.796 (1.090–2.959)	0.021	–	0. 168
C-NLR				
Low	Reference	<0.001	Reference	
High	3.171 (1.879–5.351)		2.817 (1.667–4.762)	<0.001

HR, Hazard ratio; CI, Confidence interval; AJCC, American Joint Committee on Cancer; CRP, C-reactive protein; NLR, neutrophil-to-lymphocyte ratio.

a Adjusted to age, T-stage, N-stage, M-stage, CRP, NLR, and C-NLR.

### Nomogram Development and Validation of Prognostic Efficiency

**Figure 3:**
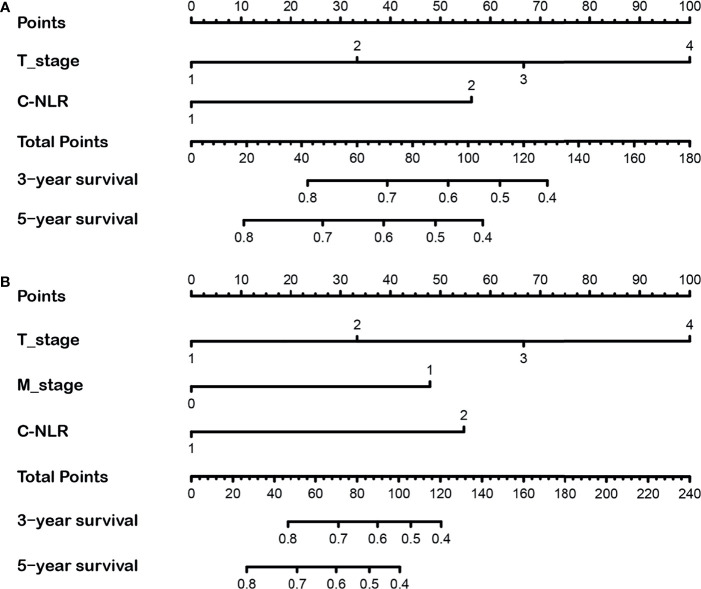
The 3- and 5-year nomograms developed by the results of the multivariate analysis for overall survival (OS) **(A)** and disease-free survival (DFS) **(B)** of bladder cancer (BC) patients after radical cystectomy.

Nomograms were developed based on the results of the multivariate analysis to quantitatively predict the OS and DFS of BC patients after RC (**Figures 3A, B**). The points of OS associated with two risk factors (T-stage and C-NLR), and DFS associated with three risk factors (T-stage, M-stage, and C-NLR). The probability of survival for BC patients receiving RC within 3 or 5 years can be predicted using the nomogram. In addition, the high area under ROC curve (AUC) of nomogram was noticed for 3-year OS, 5-year OS, 3-year DFS, and 5-year DFS, proving the nomogram had a robust predictive function for 3- and 5-year OS and DFS (**Figure S1**). Finally, as shown in **Figure S2**, high quality of calibration plots in 3- and 5-year OS and DFS nomogram models had been identified.

**Figure 4:**
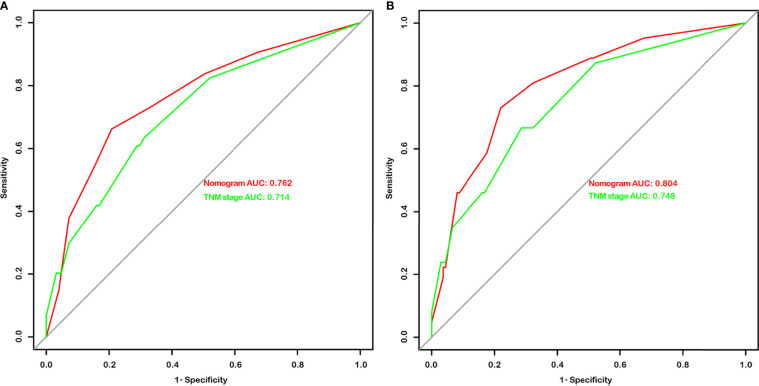
The area under the curves (AUCs) of nomograms and the TNM stage in overall survival (OS) **(A)** and disease-free survival (DFS) **(B)** prediction after radical cystectomy were compared based on the results of receiver operating characteristic (ROC) analyses.

**Figure 5:**
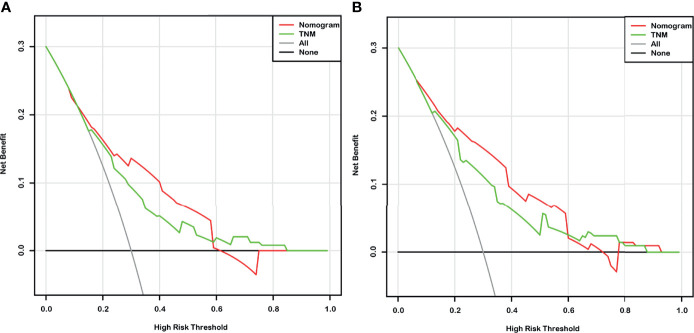
The decision curve analysis (DCA) based on nomograms and the TNM stage for overall survival (OS) **(A)** and disease-free survival (DFS) **(B)** prediction of bladder cancer (BC) patients after radical cystectomy.

Given that the TNM stage is one of the most widely used and valuable indicators for judging the clinical prognosis, we compared the clinical benefits of the nomogram and the TNM stage. ROC analyses based on nomograms were initially performed. The AUCs of nomograms of OS and DFS were 0.762 and 0.804, respectively, which were both better than the AUCs of the corresponding TNM stage, revealing that these nomograms were more powerful in predicting the prognosis of BC patients after RC compared to TNM stage (**Figures 4A, B**). Then, the DCA curves were further used to assess prognostic efficiency of the constructed nomograms, and demonstrated that the nomograms had better predictive functions for OS and DFS compared to the TNM stage (**Figures 5A, B**).

## Discussion

RC is an established standard of care for bladder cancer (12). The decision to perform RC is usually based from the imaging studies and patients’ comorbidities. However, even if “patients contain” a similar stage and grade of BC, the prognosis and clinical response may vary after RC (7). Losing the bladder is a difficult choice, and a valuable prognostic indicator for RC is certainly a vast complement to preoperative risk stratification and individual therapy. In this study, a novel predictive index C-NLR was introduced, which combines CRP and NLR.

Firstly, high C-NLR was shown to be significantly associated with some poor clinical characteristics as well as short survival times, preliminarily suggesting that C-NLR is a worthwhile metric to study. Then, we investigated the relationship between C-NLR levels and long-term outcomes of patients after RC and found that C-NLR, as an independent risk factor, was significantly associated with OS and DFS and showed superior predictive performance. Meanwhile, incorporating C-NLR and related risk factors generated nomograms predicting OS and DFS in BC patients at 3 and 5 years after RC, and compared them with classical TNM stage, validating the favorable prognostic efficiency of the nomograms. These results suggest that C-NLR is a novel and useful prognostic indicator for risk stratification and treatment planning selection for BC patients.

CRP is a predominant protein of the acute phase response and is produced in the liver (13). Its levels in the blood are associated with various inflammatory responses, including that occurring in cancer (14). With the progress of research on the relationship between tumor and inflammation, many studies on CRP or CRP-based inflammatory markers as predictors of various cancers have been reported (15–17), including urologic tumors (18). Inflammatory cells first activate pro-inflammatory cytokines such as IL-1β, IL-8, TNF-α, or IL-6, which transcriptionally activate STAT3, C/EBP, and NF-κ b pathways, leading to CRP production in the liver (10). High levels of CRP have a profound suppressive effect on adaptive immunity in cancer patients, leading to a chronic state of immune suppression and ultimately to tumor initiation and progression (19). In addition, pro-inflammatory cytokines inherently are part of angiogenesis, which influences and regulates the rise of tumor and even metastatic spread to some extent (13). This is also consistent with our findings that high CRP levels were significantly associated with lymph node metastasis. The above evidence suggests that preoperative CRP levels may shape tumor progression.

Recently, NLR has also been broadly studied as a prognostic marker for tumor patients (20–22). An increased NLR represents a relative increase in neutrophils. Neutrophils release inflammatory factors and specific proteases that induce extracellular matrix remodeling, providing a positive microenvironment for tumor cell migration and progression (23). At the same time, neutrophils can also induce apoptosis of lymphocytes leading to immunosuppression (24). A previous report by Andrea Minervini et al. also confirmed that high levels of preoperative NLR were independently associated with overall mortality (OM) in BC patients after RC (25). As we know, many blood indicators are interrelated and new tools may be needed to integrate risk factors. In this study, C-NLR consisted of CRP and NLR was proven to have stronger predictive performance than CRP and NLR in BC patients after RC. Nomograms are widely used for cancer prognosis, primarily due to their ability to reduce statistical predictive models into a single numerical estimate of the probability of an event, such as death or recurrence, that is tailored to the profile of an individual patient (26). In the present study, C-NLR-based nomograms were generated to simplify the prediction process of RC prognosis. Notably, the results of ROC and DCA showed that nomogram prediction was more effective than classical TNM staging.

Additionally, in recent years, immunomodulatory therapy using immune checkpoint inhibitors has demonstrated antitumor activity in patients with locally progressive and metastatic bladder cancer along with a favorable safety profile and durable responsiveness. The role of the immune system and inflammatory processes on clinical outcome of patients with bladder cancer is of particular interest for potential new immunotherapeutic approaches (27). Therefore, the prognostic role of C-NLR as an inflammatory index-based biomarker in bladder cancer patients using immunotherapy may be the next direction of our study.

This work firstly demonstrated that C-NLR is a reliable prognostic indicator for BC patients who underwent RC and aids in preoperative risk stratification and individual therapy. Nonetheless, there are still potential limitations including single-center retrospective study and a small sample size. A previous study has confirmed that postoperative NLR and CRP levels are also associated with the survival of patients after RC (28), whereas the laboratory data in our study were taken from a single preoperative assessment, and their changes over time and response to treatment have not been evaluated. In addition, although every effort has been made to control potential confounders, there may still be individual undocumented factors influencing inflammatory markers in some patients. Therefore, subsequent prospective multicenter studies are still needed to validate our results.

## Conclusion

C-NLR is a novel, readily available, and widely applicable prognostic index for BC patients after RC. Our results aid in optimizing the individual therapy. The nomogram is a credible model for predicting 3- and 5-year OS and DFS of patients after RC.

## Data Availability Statement

The raw data supporting the conclusions of this article will be made available by the authors, without undue reservation.

## Ethics Statement

The studies involving human participants were reviewed and approved by Ethics Committee of Shanghai Tenth People’s Hospital, School of Medicine, Tongji University (SHSY-IEC-KY-4.0/18-68/01). The patients/participants provided their written informed consent to participate in this study.

## Author Contributions

Conception and design: YW, WM, and BP. Administrative support: BP. Provision of study materials or patients: JN and HZ. Collection and assembly of data: NZ, LY, and YZ. Data analysis and interpretation: YW, KW, and JN. Manuscript writing: All authors. All authors contributed to the article and approved the submitted version.

## Funding

This work was supported by the National Natural Science Foundation of China (Grant Nos. 81870517 and 32070646), the Shanghai Association for Science and Technology Commission (Grant No. 19140905700), and the Science and Technology Innovation Project of Putuo District Health Commission (Grant No. In review ptkwws201916).

## Conflict of Interest

The authors declare that the research was conducted in the absence of any commercial or financial relationships that could be construed as a potential conflict of interest.

## Publisher’s Note

All claims expressed in this article are solely those of the authors and do not necessarily represent those of their affiliated organizations, or those of the publisher, the editors and the reviewers. Any product that may be evaluated in this article, or claim that may be made by its manufacturer, is not guaranteed or endorsed by the publisher.

## Supplementary Material

Supplementary Figure 1 The receiver operating characteristic (ROC) analyses curves based on the nanogram for the 3- and 5-year overall survival (OS) **(A, C)** and disease-free survival (DFS) **(B, D)** prediction.

Supplementary Figure 2 Calibration plot of the 3- and 5-year overall survival (OS) **(A, C)** and disease-free survival (DFS) **(B, D)** nomograms.
