# Supplementary figures and images for: Combination of C-Reactive Protein and Neutrophil-to-Lymphocyte Ratio as a Novel Prognostic Index in Patients With Bladder Cancer After Radical Cystectomy
Source: Front Oncol. 2021 Dec 2;11:762470. doi: 10.3389/fonc.2021.762470 (PMC8674495; doi:10.3389/fonc.2021.762470)

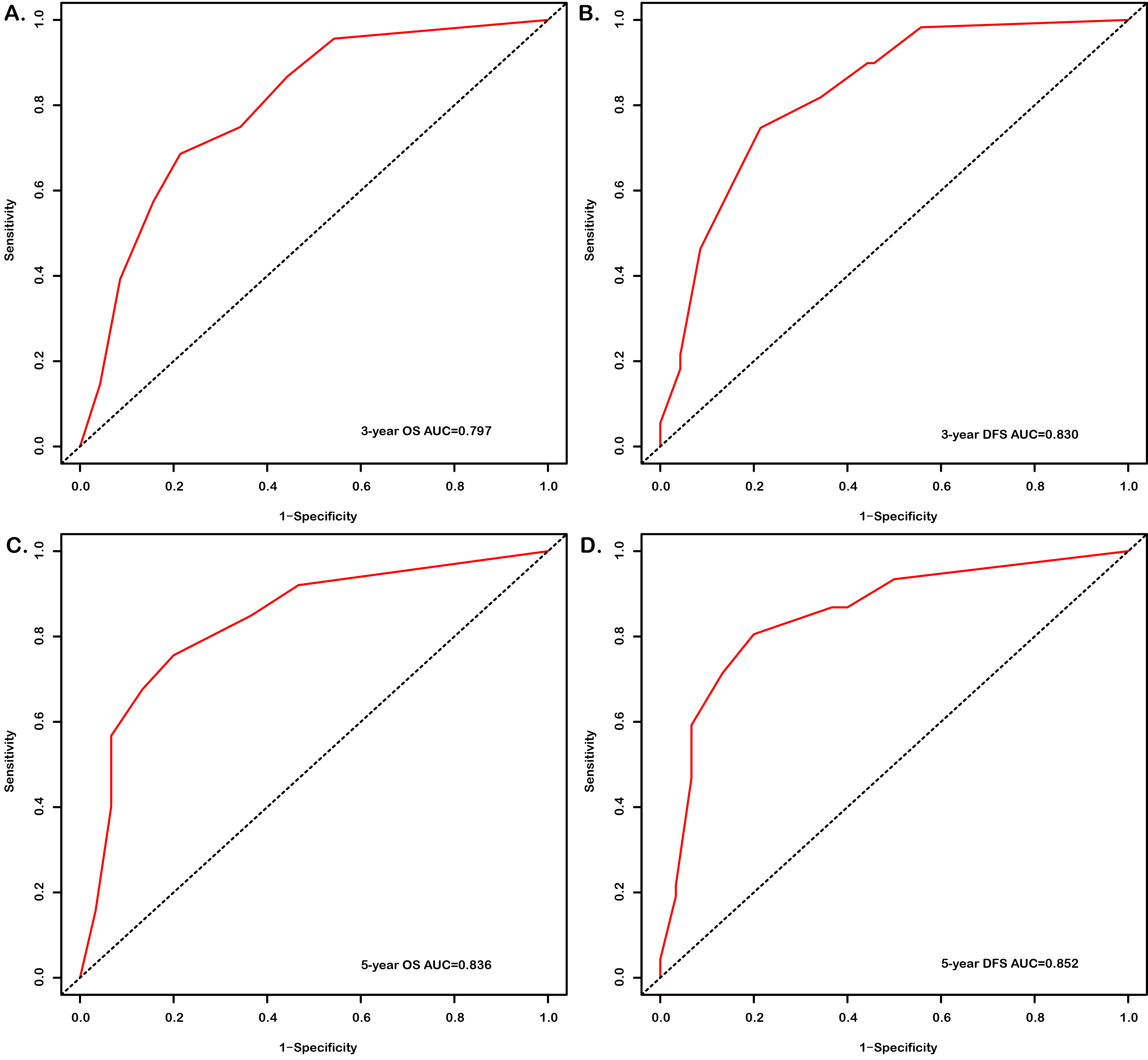

Supplement: Supplementary Figure 1 — The receiver operating characteristic (ROC) analyses curves based on the nanogram for the 3- and 5-year overall survival (OS) (A, C) and disease-free survival (DFS) (B, D) prediction. [file Image_1.tif]

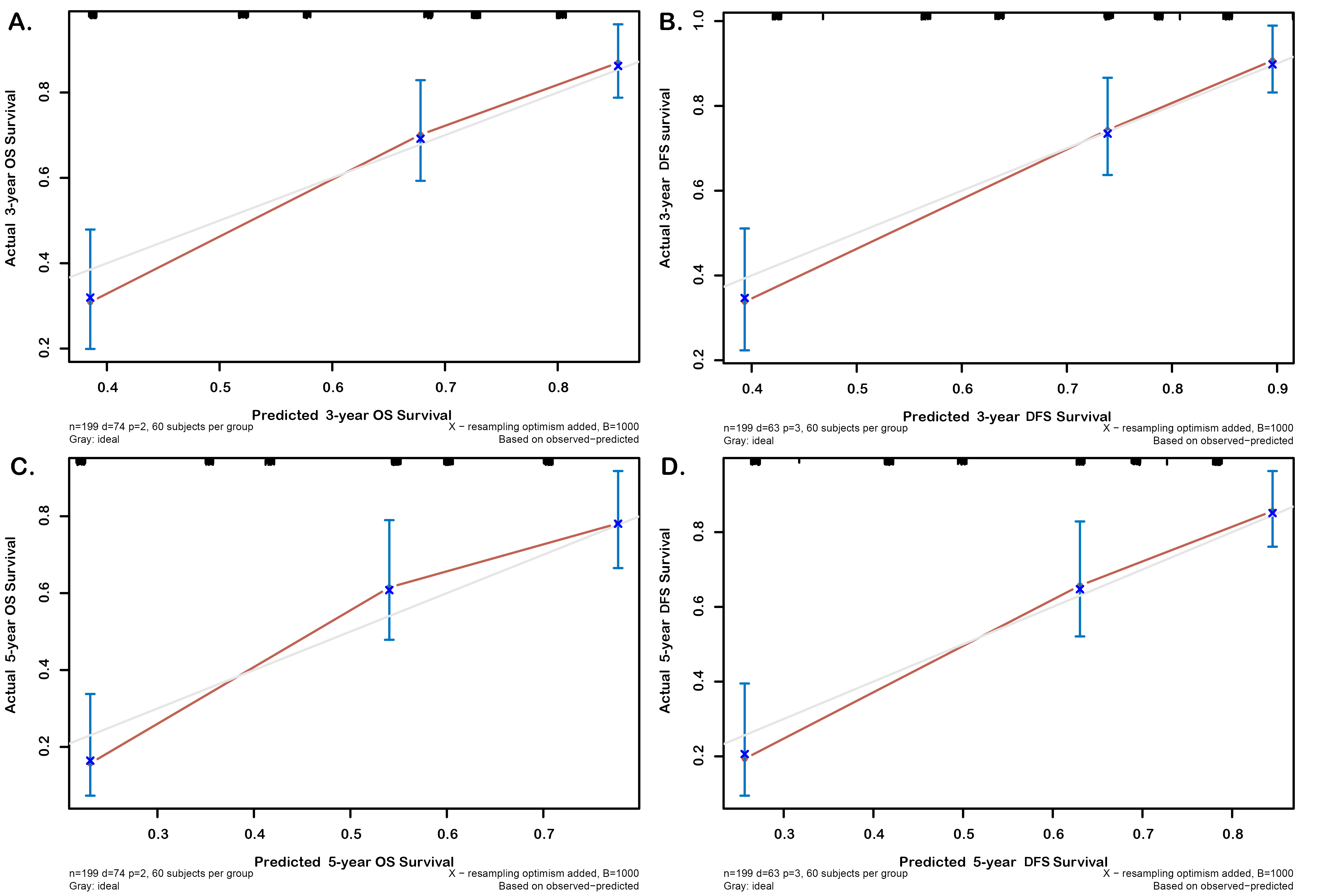

Supplement: Supplementary Figure 2 — Calibration plot of the 3- and 5-year overall survival (OS) (A, C) and disease-free survival (DFS) (B, D) nomograms. [file Image_2.tif]
